# Reduction of protein kinase C α (PKC-α) promote apoptosis *via* down-regulation of Dicer in bladder cancer

**DOI:** 10.1111/jcmm.12503

**Published:** 2015-03-05

**Authors:** Zhenming Jiang, Chuize Kong, Zhe Zhang, Yuyan Zhu, Yuxi Zhang, Xi Chen

**Affiliations:** a Department of Urology, The First Affiliated Hospital of China Medical UniversityShenyang, China; b Department of Pharmacology, The First Affiliated Hospital of China Medical UniversityShenyang, China

**Keywords:** PKC-α, Dicer, apoptosis, bladder cancer, caspase-3

## Abstract

In clinic, we examined the expression of protein kinase C (PKC)-α and Dicer in the samples of bladder cancer patients, and found that the two proteins have a line correlation. Our study confirmed this correlation existing by clearing the decreasing expression of Dicer after the PKC-α knockdown. Treatment of bladder cancer cell lines (T24, 5637) with the PKC-α or Dicer knockdown and the PKC inhibitors (Calphostin C and Gö 6976) can promote the apoptosis. Inhibition of PKC can increase the activities of caspase-3 and PARP, however, decrease the expression of Dicer. And knockdown of the PKC-α or Dicer can also activate the caspase-3 or the PARP. Considering the reduction of PKC-α can induce the Dicer down-regulation, we make the conclusion that the reduction of PKC-α can promote the apoptosis *via* the down-regulation of Dicer in bladder cancer.

## Introduction

Bladder cancer (BC) is one of the most recurrent malignant tumours and also the second most common urologic cancer 1. In the US, BC is the ninth most common cause of cancer-related mortality, and is the fourth most common cancer in men 2. As the central hub of many signal transduction process, protein kinase C (PKCs) are involved in various cellular processes such as regulation of gene expression, proliferation, cell junctions, apoptosis, and migration. PKC family consists of serine-threonine kinases that act by phosphorylating their specific protein substrates. The family members are classified into three groups: cPKCs (classical PKCs, including α, β, and γ); nPKCs (novel PKCs, including δ, ε, η, and θ); and aPKCs (atypical PKCs, including μ, ξ) 3. In the current studies, many agents and factors can modulate the apoptosis of carcinoma cells *via* protection or inhibition of the PKC-α 3. But the apoptotic mechanism of PKC-α in BC remains unclear until now.

Dicer is one of the ribonuclease III enzymes renowned for its kernel role in the biogenesis of microRNAs (miRNAs) 4. Because of the only copy of Dicer in the human being's genome, inhibiting or knockdown of it should theoretically produce cells that are deficient in miRNAs. Array expression profiling analysis has revealed a global reduction of miRNAs expression in various cancer models. This observation led to a hypothesis that the low expression of Dicer could be the key factor in tumour tissue initiation or program death. In addition, C-terminal fragment of Dicer also possesses DNase activity that is critical for DNA fragmentation during apoptosis 5. Thus, we think the Dicer should be a critical factor in the BC apoptosis.

Here, we show that the direct correlation between the PKC-α and Dicer. After we have the PKC-α and Dicer inhibition and/or knockdown, the apoptosis of two BC cell lines rise synchronously. Moreover, the mechanism of the apoptosis occurs through the same classic caspase-3-PARP pathway. Our results point to another pathway of PKC-α regulation of apoptosis and suggest that Dicer can be involved in this process.

## Materials and methods

### Tissue samples

39 tissue samples of BC were collected from patients underwent transurethral resection of bladder tumour (TUR-Bt) or radical cystectomy in the Department of Urology at the First Affiliated Hospital of China Medical University. And we also collected six normal urothelium samples, which were cut-off 1.5 cm away from the tumour margin among the patients underwent the cystectomy. All the surgeries occurred between October 2012 and October 2013, and all patients provided signed informed consent. Immediately following resection, the samples of carcinoma were placed in −80°C storage until the time of RNA extraction.

### Cell culture and transfection of siRNAs

Human urinary bladder carcinoma cell lines, T24 and 5637, immortalized human bladder epithelium SV-HUC-1 (SV) cell were propagated in RPMI 1640 medium supplemented with 10% (v/v) foetal bovine serum, 100 U/ml penicillin, and 100 μg/ml streptomycin at 37°C in a humidified atmosphere of 95% air and 5% CO_2_. siRNAs against PKC-α and DICER were purchased from Genepharma (Shanghai, China). siRNAs were transfected into cells with Lipofectamine TM 2000 (Invitrogen, Life Technologies, Carlsbad, CA, USA) according to the manufacturer's instruction. And the medium was changed in 6–8 hrs. Silencing efficiency was evaluated by real-time PCR analysis and Western-blotting analysis, 24–48 hrs after the medium changing.

### Antibodies

Rabbit polyclonal antibody against PARP (sc-25780), mouse polyclonal antibodies against PKC-α (sc-8393) and DICER (sc-136981) were purchased from Santa Cruz Biotechnology, Inc. (Dallas, Texas, USA). Mouse polyclonal antibody against GAPDH was purchased from Sigma Aldrich Biotechnology (St. Louis, MO, USA).

### Western-blotting analyses

Cells and tissue samples were lysed in RIPA buffer (20 mM Tris/HCl, pH 7.4, 150 mM NaCl, 1% Triton X-100, and 0.5% sodium deoxycholate, 0.1% SDS, 2 mM EDTA and 1 mM dithiothreitol) for 30 min. on ice. Lysates were cleared by centrifugation for 35 min. at 7000 × g, and protein contents were estimated employing BCA reagent (Beyotime, Shanghai, China). Equal amounts of protein (50 μg for cell lysate) were separated by 8–10% SDS/PAGE and blotted on to PVDF membranes. The membranes were exposed to blocking reagent (no fat milk/TBST), and subsequently incubated overnight at 4°C with the primary antibodies, followed by rabbit or mouse peroxidase-conjugated secondary antibodies. Quantification of the proteins was done by normalization to GAPDH and expressed as arbitrary units.

### Quantitative real-time PCR analysis

Total RNA was isolated from cultured cells and tissue with 4°C-cold TRIZOL reagent (Invitrogen, Life Technologies, Carlsbad, CA, USA), according to the manufacturer's protocol. The concentration of RNA was determined by Thermo Scientific NanoDrop ND-100 (Wilmington, DE, USA), and then, reverse transcription of 2 μl of total RNA was performed with SYBR® PrimeScript® RT-PCR Kit (Perfect Real Time; Takara, Kyoto, Japan). Real-time PCR analysis of the cDNA was quantified using LightCycler 480 (Roche Diagnostics GmbH, Roche Applied Science, Mannheim, Germany). The reaction system was kept at 95°C for 30 sec. Subsequently denaturing the mixture at 95°C for 5 sec., annealing at 55°C for 30 sec., and this step repeated 45 cycles. The primer sequences are as follows: β-actin: sense: 5′-TTGGCAATGAGCGGTTCCGCTG-3′, anti-sense: 5′-TACACGTGTTTGCGGATGTCCAC-3′; PKC-α: sense: 5′-GGAACCACAAGCAGTATT-3′, anti-sense: 5′-GTCCTTCTGAATCCAACAT-3′; Dicer: sense: 5′-GATTCTGAGGATGATGATGAG-3′, anti-sense: 5′-CAACTGCTGTGTATCTTCTT-3′.

### Quantitative detection of apoptosis by flow cytometry

To determine the apoptosis in T24 and 5637 cell lines, the Annexin V–FITC/PI Apoptosis Detection kit (Keygen, Nanjing, China) was used following the protocol. A total of 1 × 10^6^ cells were seeded onto 6-well plates and treated with SiRNA against PKC-α or DICER for 6 hrs. After 24 hrs, cells were collected by trypsinization and washed with cold-PBS, resuspended in the 200 μl Binding Buffer; the cells were stained with Annexin V-FITC and propidium iodide (PI) for 15 min. in the dark at room temperature following the protocol provided by the manufacturer. FACScan flow cytometer and Cell-Quest software (Becton-Dickinson, Bedford, MA, USA) were used to analyse the apoptotic cells.

### Protein kinase C inhibitors

Protein kinase C function inhibition in the two carcinoma cell lines were induced with 500 nM of Gö 6976 (Sigma-Aldrich, St. Louis, MO, USA) and 50 nM of Calphostin C (Sigma-Aldrich, St. Louis, MO, USA).

### Immunocytochemistry

To determine the localization variation of Dicer, cells growing on 24-well chamber slides were fixed with 4% paraformaldehyde and permeabilized with 0.5% Triton-X100. Fixed cells were then probed with the anti-Dicer antibody overnight at 4°C after blocking with 5% normal bovine serum for 1 hr. They were then treated with an Alexa Fluor 488-conjugated secondary antibody for 1 hr. Nuclear staining was performed with DAPI (Beyotime).

### Caspase-3 activity analysis

To determine the caspase-3 activity of the two bladder carcinoma cells, the caspase-3 colorimetric assay kit (Keygen) was used following the protocol. The cells were seeded in the 25 cm^2^ flask, then propagated in PRMI 1640 medium with PKC-α inhibitors, or transfected by the siRNAs against PKC-α and Dicer. And we use the Lysis Buffer in the kit to collect the cells with inhibitors at 0, 8, 16 and 24 hrs; and the cells with transfection was collected after 24 hrs. And protein contents were estimated employing Bradford reagent, equal amount protein were stained with caspase-3 Substrate in 37°C for 4 hrs. Spectrophotometer was used to analyse the caspase-3 activity at 405 nm.

### Statistics analysis

Data were expressed as the mean ± SD for three independent experiments. Differences between samples were analysed by independent sample *t*-tests using SPSS V20.0 software (IBM Corp., Armonk, NY, USA). Correlation between two groups was analysed using bivariate correlation analysis. Statistical significance was designated at *P* < 0.05 as compared with the corresponding control or as specifically indicated.

## Results

### The PKC-α and Dicer have correlative relationship in human BC specimens and decreasing the PKC-α can down-regulate the expression of Dicer in BC cells

**Figure 1:**
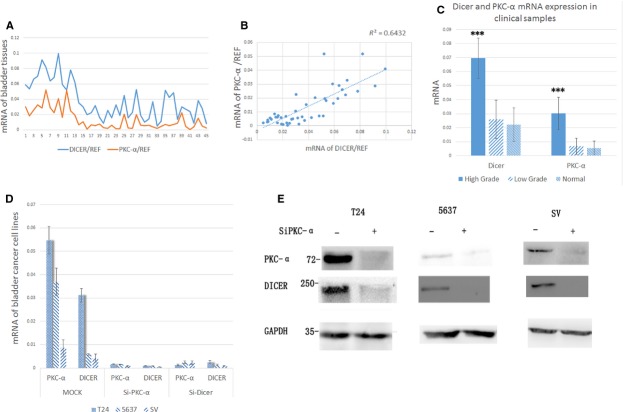
(A) The real-time PCR analysis of the PKC-α and Dicer expression in 45 bladder cancer samples and six normal urothelium samples. (B) Correlative relationships through 45 samples. Scatterplots and correlation analysis of expression profiling data as determined by real-time PCR for PKC-α to Dicer as shown. *r*^2^ = 1 indicates perfect correlation. (C) Dicer and PKC-α mRNA expression in the clinical samples which were divided into three groups, including high-grade bladder cancer, low-grade bladder cancer and normal tissues. Each expression level was shown as mean ± SD. (D) The real-time PCR analysis of the PKC-α and Dicer expression in T24, 5637 and SV-HUC-40 cell lines. (E) The western blotting analysis of the PKC-α and Dicer expression in T24, 5637, and SV-HUC-40 cell lines. (D and E) The experiments were repeated for over three times and the similar results were obtained. *P < 0.05; **P < 0.01; ***P < 0.001.

We examined the expression level of PKC-α and Dicer in the 39 primary human BC samples and 6 normal urothelium samples, using real-time PCR analysis. Figure1A shows that although the expression level of PKC-α doesn't always correspond to that of the Dicer, all the samples have the tendency to the correlation between the both. Then we used the scatterplots and correlation analysis to confirm the relation (Fig.1B). We found that the expression of them have a strong correlation (*r* = 0.80, *P* < 0.01). This result shows that the two proteins may have some interaction or signalling pathway. Furthermore, real-time PCR results of Dicer and PKC-α expression in clinical BC samples showed that the both proteins were produced more in the high-grade papillary urothelial carcinoma (HGPUC) than in the low-grade papillary urothelial carcinoma (LGPUC; Dicer: <0.01, PKC-α: <0.01)or in the normal urothelium (Dicer: <0.01, PKC-α: <0.01). However, compared with the normal urothelium, we did not observe the significant difference in the LGPUC samples (Dicer: 0.54, PKC-α: 0.59; Table1). We subsequently do the further research in the BC cell lines.

**Table 1:** 

Parameters	Cases	*P*-value	
		Dicer-mRNA	PKC-α-mRNA
Overall	39		
Gender			
M	31	0.551	0.41
F	8		
Age at surgery			
>55	30	0.488	0.515
≤55	9		
Muscle invasive			
Invasive	6	0.001***	0.001***
Non-invasive	33		
Grade			
Normal	6	HGPUC compared Normal: <0.01**	HGPUC compared Normal: <0.01**
LGPUC	26	LGPUC compared Normal: 0.54	LGPUC compared Normal: 0.59
HGPUC	13	HGPUC compared LGPUC: <0.01**	HGPUC compared LGPUC: <0.01**
Metastasis			
Absent	39		
Present	0		
Lymphatic permeation			
Absent	39		
Present	0		

* *P* < 0.05

** *P* < 0.01

*** *P* < 0.001.

We chose two BC cell lines, T24 and 5637, and one immortalized bladder epithelial cell line, SV, for the further research. T24 is a kind of BC cell line originated from a Swedish 82-year-old female in 1970, which is a Grade-III carcinoma 6. 5637, which is a Grade-II carcinoma, was originated from a 68-year-old male in 1974, and can secrete some cytokines, including growth factor, G-CSF, GM-CSF, IL-3, *etc* 7. We transfected the three cell lines with siRNAs against the PKC-α or Dicer, and designated as siPKC-α and siDicer. Mock (siRNA against null) served as a control. Then we examined the expression changes of them separately, using the real-time PCR analysis and western blotting analysis. Figure1D and E showed when we interfered the production of the PKC-α, the expression of Dicer would be down-regulated synchronically. So we consider the PKC-α can modulate the Dicer transcription or expression, though Figure1D figured out that knocked off the Dicer can also induce the PKC-α low expression. And we will discuss this topic later.

### Knockdown of PKC-α and Dicer can promote the apoptosis of BC

**Figure 2:**
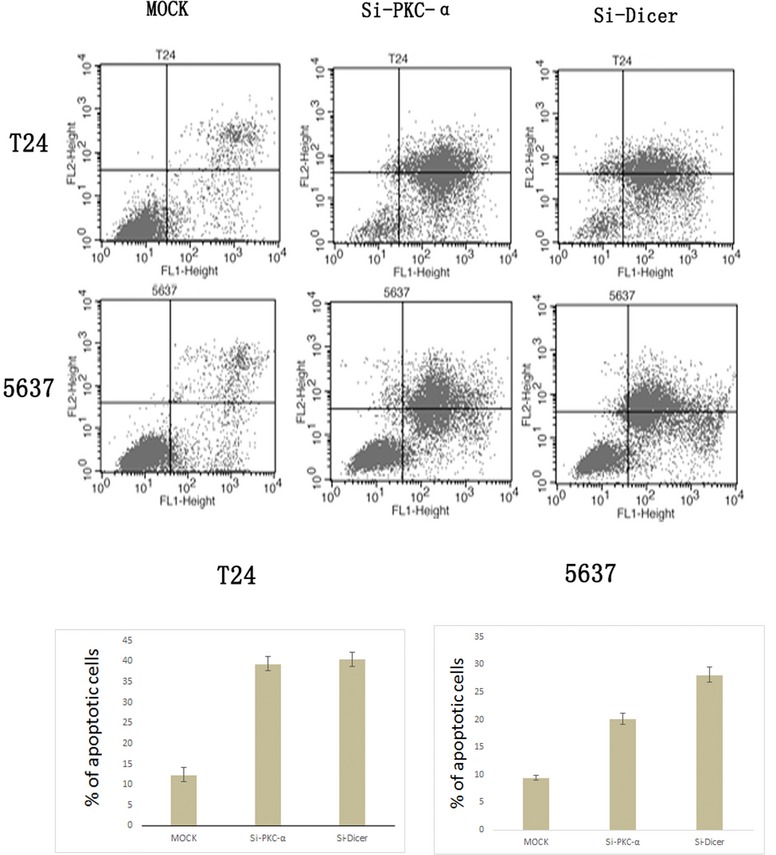
T24 and 5637 cell, with transducing mock SiRNA (MOCK) or with transducing PKC-α SiRNA (Si-PKC-α) or with transducing Dicer SiRNA (Si-Dicer), were seeded on the equal density. The cells were stained by the Annexin-V/PI after equal condition culturing, and subjected to the FACS for apoptotic analysis (left Panels). The early apoptosis cells (Annexin-V positive, Q4 cells) were plotted into the bar graph (right panels). These experiments were repeated for three times and the similar results were obtained.

To determine if the PKC-α and Dicer contribute to the apoptosis of the BC, we inoculated T24 and 5637 BC cells with or without PKC-α and Dicer knockdown, using FACS subsequently to detect the apoptosis cells. We found that the two BC cell lines with PKC-α and Dicer knockdown were more sensitive to the apoptosis than those with mock knockdown after 24 hrs cultured respectively (Fig.2, phase Q4). After we knock off the PKC-α and the Dicer in T24, the mean percentage of apoptotic cells had increased from 12.42% to 39.37% and 40.53% respectively. And in the 5637 cells, the numbers had increased from 9.49% to 20.22% and 28.13% respectively. These results showed that the lack of either PKC-α or Dicer can promote the apoptosis of the two BC cell lines.

### The involvement of caspase-3-PARP pathway in the PKC-α or Dicer induced apoptosis

**Figure 3:**
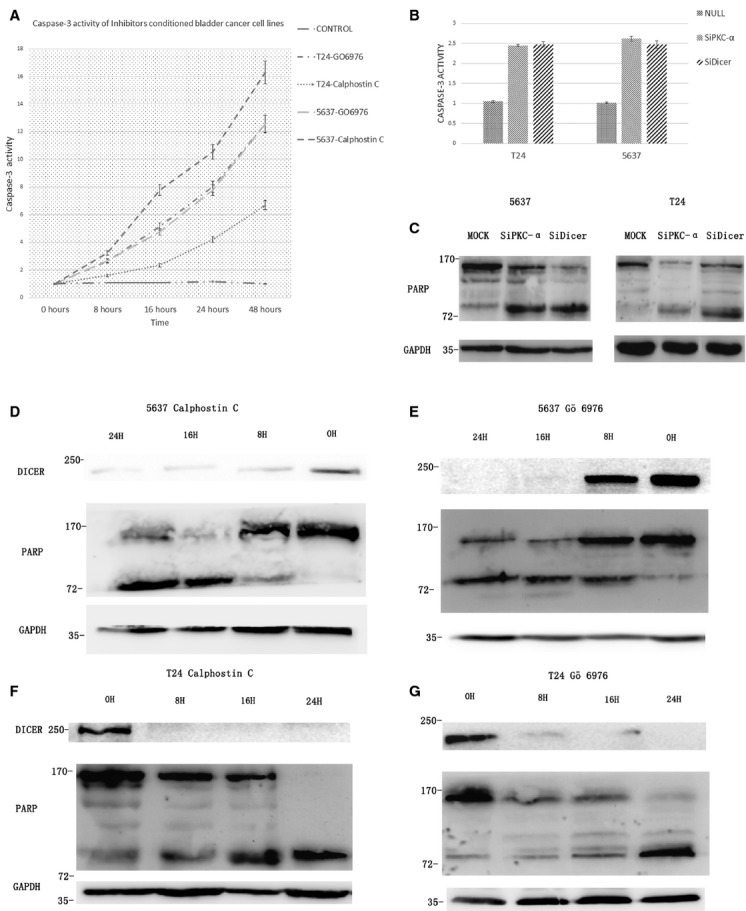
(A) T24 and 5637 cells were incubated with Calphostin C and Go 6976. At indicated time-point (hours) cells were lysed and lysates were analysis by caspase-3 activity analysis kit. (B) T24 and 5637 cells were transfected by the mock, Si-PKC-α and Si-Dicer for 6 hrs, and the cells were washed and fresh medium were added. Cells were lysed 24 hrs later and the lysates were analysed by caspase-3 activity analysis kit. (C) T24 and 5637 cells were transfected by the mock, Si-PKC-α and Si-Dicer for 6 hrs, and the cells were washed and fresh medium were added. Cells were lysed 24 hrs later, the lysates were analysed by western blotting. (D–G) 5637 (D and E) and T24 (F and G) were incubated with Calphostin C (D and F) and Go 6976 (E and G) for indicated time (hours) and the cells were lysed by the RIPA and the lysates were analysed with western blotting. *P < 0.05; **P < 0.01; ***P < 0.001.

To confirm that the increased rate of cell apoptosis was due to caspase-3, we tested the activity of caspase-3 in the cells with the PKC-α and Dicer knockdown and PKC inhibitors (Calphstin C and Gö 6976) treatment, using the caspase-3 colorimetric assay kit. The results show that the longer time we incubated T24 and 5637 cell lines with the two inhibitors, the higher level of the caspase-3 activities possessed (Fig.3A). And in the cases of siPKC-α and siDicer transfected cells were undergoing accelerated caspase-3 activities compared with the control MOCK (transfected void vector) cells (Fig.3B). Another hallmark of apoptosis is the caspase-mediated cleavage of the nuclear 116 kD protein PARP to a smaller 85 kD inactive form 8. Western blotting analysis reveals an increased cleavage of PARP in the siPKC-α and siDicer cells (T24 and 5637) compared with the MOCKs (Fig.3C). Furthermore, incubation of the two BC cells with the two PKC inhibitors can resulted in the Dicer down-regulation and significant cleavage of PARP. And the levels of the Dicer down-regulation and cleavage of PARP both have the time dependence (Fig.3D–G). These results showed that the apoptosis-related down-regulation of the Dicer may require the inhibition of PKC-α. Importantly, the decreased amount of PKC-α or Dicer can contribute to accelerated apoptosis in the same caspase-3-PARP pathway. In addition, the decreased amount of PKC-α can induce the Dicer down-regulation. Thus, it is reasonable that the Dicer should be an important agency of the PKC-α induced BC cell apoptosis.

### The location of Dicer expression has no change after PKC-α knockdown in T24, 5637 and SV cell lines

**Figure 4:**
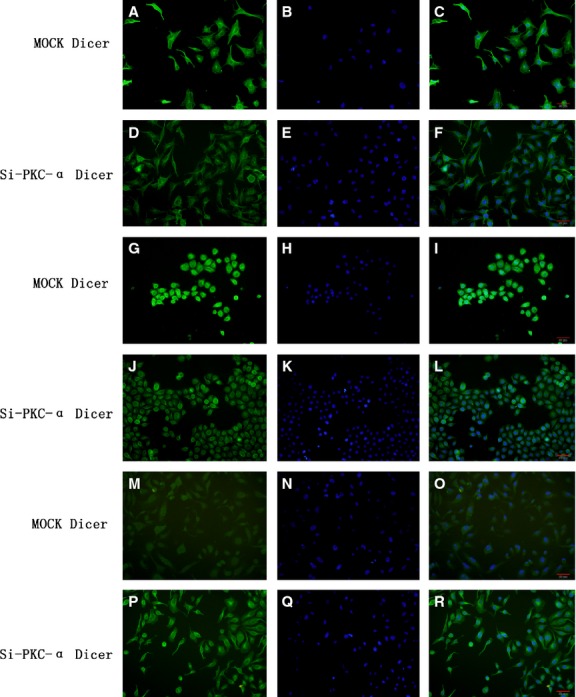
Location of Dicer in the T24, 5637 and SV cell lines detected by the immunofluorescence. The letters represented the following cells: (A–F) T24 cells, (G–L) 5637 cells, (M–R) SV cells. Dicer (green) were observed in (A, D, G, J, M and P); nuclear staining was performed DAPI (blue) in (B, E, H, K, N and Q). The merged panels were (C, F, I, L, O and R). The panel (A–C, G–I and M–O) illustrated the cells transfected mock; and the panel (D–F, J–L and P–R) illustrated the cells transfected PKC-αSiRNA.

The immunofluorescence was performed to confirm the location of Dicer expression in T24, 5637 and SV cell lines. As shown in Figure4A–R, the location of the Dicer expression in the PKC-α knockdown cells, has no change compared with that in the MOCK cells. The Dicer was mainly found to work in the cytosol, especially highlight around the cell nuclear.

## Discussion

In this study, we discovered evidence supporting a critical role for PKC-α-Dicer signalling pathway in human BC apoptosis. Firstly, we found the positive linear correlation between PKC-α and Dicer in 45 bladder samples (including 39 tumours and 6 normal urothelium). Secondly, on the basis of this result, we tried to knock off the PKC-α in two BC cell lines and one immortality bladder epithelial cell line, to confirm the correlation, and found that the down-regulation expression of the PKC-α can induce the lower expression of the Dicer in mRNA and protein levels. Thirdly, we also decreased the expression of the Dicer, and found that the PKC-α was down-regulated, either. However, considering the Dicer whose function is essential for the maturation of almost all microRNAs 9. It should be reasonable that the lack of Dicer induced PKC-α down-regulation is the conclusion of lack of miRNAs. On the other hand, the function of PKC-α is considered as the central hub of various signalling transduction progress 10. So we believed that the decreased number of PKC-α should be the origin that the Dicer was down-regulated synchronically. Lastly, through the interfering PKC-α expression, the Dicer's mRNAs formation was suppressed. This showed that PKC-α might initiate some signalling factors, entering the nucleus to modulate the Dicer's transcription. However, the exact signalling pathway is a mystery tour which should be revealed by further researches. And we'd like to provide two possibilities of the pathway in the following paragraphs.

Down-regulation of Dicer was reported to induce apoptosis in Hela cell line 11 and prostate cancer 12. In addition, decreased level of Dicer expression was found to be related to advanced tumour stages or poor clinical outcome in melanoma 13, neuroblastoma 14, breast 15, lung 16, and ovarian cancers 17, and some PKC inhibitors can reach a similar conclusion. Especially, we found that the mRNAs of the Dicer and the PKC-α were formed more in the HGPUC than in the LGPUC or in the normal tissue. And it is reasonable that the comparatively low level of the two proteins might block the malignant tendency of the bladder tissues. So we thought there should be some effect on the bladder carcinoma apoptosis. In our study, we stepped further about this story. FACS apoptosis assay confirmed that the PKC-α and the Dicer knockdown can induce the apoptosis separately in the two BC cells (T24, 5637). Interestingly, the percentage of apoptotic cells of T24 cell lines are more than that of 5637 group. We think that this protection effect might involve in the various cytokines secretion of 5637.

Subsequently, we focused on the underlying apoptosis mechanism. Activation of caspase-3 and following cleavage of its substrates such as PARP are the hallmarks of apoptosis. Following the two kinds of PKC inhibitors (Calphostin C and Gö 6976) conditioned cell lines; we found the time dependent changes between the PARP-cleavage apoptosis pathway and the inhibition of Dicer. These results showed that the PARP-cleavage during apoptosis can be controlled by Dicer. And the PKC isoforms should be involved in this process. Furthermore, we investigated the apoptosis of PKC-α and Dicer knocking-down transcription, and found that either of them induced the apoptosis through the same caspase-3-PARP pathway, which is similar to the above. According to these findings, including the PKC-α-Dicer relationship and the identical caspase-3-PARP apoptosis pathway, we deduced that the Dicer should be a critical role in the PKC-α induced apoptosis.

Using the immunofluorescence, we found that the Dicer was located in the cytosol, especially surrounding the nuclear. The location of PKC-α is also in the cytosol 18. Because the PKC-α cannot enter nuclear 19, we think it should initiate some signalling pathway, which will be our subsequent concern. Increasing studies are considering such things, especially about the Dicer regulation aspect, in these 2 years. Among these studies, there are two pathways, in my opinion, should be paid more attention to. We would like to discuss the P53-P63-Dicer pathway firstly. Muller and his crew 20 found that the mutant P53 can regulate Dicer expression through the TAp63-dependent and TAp63-independent mechanisms. Considering about the interwoven links of the PKC-α and P53 21,22, we have reason to believe that P53 could play an important role in the PKC-α exerting influence on Dicer. Secondly, the let-7 can regulate the Dicer expression 23. And the PKC-α can exert influence on the YAP which is a key protein in the HIPPO pathway 24. Moreover, the HIPPO pathway also exerts the regulation function on the Dicer expression through the TAZ/YAP-let-7 pathway 25. So, the HIPPO-let-7 mechanism can also bridge the gap between the PKC-α and the Dicer. Although there are many untapped virgin lands about the mechanism of PKC-α affecting Dicer expression, we are sure that more researches will focus on this aspect in the near future.

In summary, the lower expression of PKC-α can decrease the expression of Dicer, and activate the caspase-3-PARP apoptosis pathway, finally contribute to progression of apoptosis in human BC.
